# Predicting COPD exacerbations based on quantitative CT analysis: an external validation study

**DOI:** 10.3389/fmed.2024.1370917

**Published:** 2024-06-12

**Authors:** Ji Wu, Yao Lu, Sunbin Dong, Luyang Wu, Xiping Shen

**Affiliations:** ^1^Department of General Surgery, Suzhou Ninth Hospital Affiliated to Soochow University, Suzhou, China; ^2^Department of Anesthesia, Fifth People's Hospital of Wujiang District, Suzhou, China; ^3^Department of General Medicine, Municipal Hospital, Suzhou, China

**Keywords:** chronic obstructive pulmonary disease, image analysis, deep learning system, body composition, skeletal muscle

## Abstract

**Purpose:**

Quantitative computed tomography (CT) analysis is an important method for diagnosis and severity evaluation of lung diseases. However, the association between CT-derived biomarkers and chronic obstructive pulmonary disease (COPD) exacerbations remains unclear. We aimed to investigate its potential in predicting COPD exacerbations.

**Methods:**

Patients with COPD were consecutively enrolled, and their data were analyzed in this retrospective study. Body composition and thoracic abnormalities were analyzed from chest CT scans. Logistic regression analysis was performed to identify independent risk factors of exacerbation. Based on 2-year follow-up data, the deep learning system (DLS) was developed to predict future exacerbations. Receiver operating characteristic (ROC) curve analysis was conducted to assess the diagnostic performance. Finally, the survival analysis was performed to further evaluate the potential of the DLS in risk stratification.

**Results:**

A total of 1,150 eligible patients were included and followed up for 2 years. Multivariate analysis revealed that CT-derived high affected lung volume/total lung capacity (ALV/TLC) ratio, high visceral adipose tissue area (VAT), and low pectoralis muscle cross-sectional area (CSA) were independent risk factors causing COPD exacerbations. The DLS outperformed exacerbation history and the BMI, airflow obstruction, dyspnea, and exercise capacity (BODE) index, with an area under the ROC (AUC) value of 0.88 (95%CI, 0.82–0.92) in the internal cohort and 0.86 (95%CI, 0.81–0.89) in the external cohort. The DeLong test revealed significance between this system and conventional scores in the test cohorts (*p* < 0.05). In the survival analysis, patients with higher risk were susceptible to exacerbation events.

**Conclusion:**

The DLS could allow accurate prediction of COPD exacerbations. The newly identified CT biomarkers (ALV/TLC ratio, VAT, and pectoralis muscle CSA) could potentially enable investigation into underlying mechanisms responsible for exacerbations.

## Introduction

Chronic obstructive pulmonary disease (COPD) is a global health problem. Acute exacerbations were defined as acute worsening of respiratory symptoms associated with lung function decline and long-term outcomes (1, 2). COPD exacerbations are characterized by an acute deterioration in respiratory symptoms. This event requires additional therapy and further increases the economic burden on patients (3). COPD exacerbations may be attributed to many risk factors, such as respiratory viral infections, the exacerbation of other respiratory diseases, and non-respiratory diseases (4). Some individuals can progress to respiratory failure, requiring intensive care and eventually death, for example, patients with certain comorbidities (5). To the best of our knowledge, early-risk stratification of COPD patients and taking supportive care measures are central to avoiding the worst progression and fatal outcomes (6). Some risk stratification scores, such as the BMI, airflow obstruction, dyspnea, and exercise capacity (BODE) index, have been proposed to predict COPD exacerbations in COPD patients (7–10). However, these scores have several limitations, such as low AUC values (11).

Deep learning (DL) methods are being increasingly integrated into scientific discovery (12–14). Nowadays, the DL algorithm plays a pivotal role in data mining and analysis of medical imaging. Reportedly, the DL algorithm can be used to evaluate the severity of emphysema using sequential CT scans (15). Additionally, the DL algorithm also performed well in the prediction of hospital readmissions and long-term prognosis for COPD (16, 17). Notably, these algorithms also showed excellent performance in auxiliary diagnosis of infectious diseases, such as sepsis (18). However, studies evaluating the capacity of DL techniques in risk stratification of COPD patients are limited.

Relevant information on body composition and thoracic abnormalities can be obtained by quantitative computed tomography (CT) analysis (19–21). CT imaging features have been used to identify individuals at risk of progressing to COPD (22). Reportedly, Moll et al. developed a tool for predicting all-cause mortality based on quantitative CT imaging features in patients with COPD (23). Shimizu et al. (24) devised a model for predicting a decline in lung function and mortality in COPD using the chest inspiratory CT scan. Although the clinical potential of CT biomarkers has been explored, the role of CT scan-derived indicators in predicting COPD exacerbations remained unclear.

Hence, we aimed to assess the added potential of CT biomarkers in predicting COPD exacerbations and thus develop and externally validate a DL model.

## Methods and materials

### Study design

In this retrospective study, hospitalized patients with COPD were consecutively recruited at Suzhou Ninth People’s Hospital of Soochow University between January 2013 and March 2021. The inclusion criteria were as follows: (1) age ≥ 18 years old, (2) confirmed by pulmonary function tests at the baseline visit (post-bronchodilator FEV1/FVC ratio less than 0.7), (3) receiving chest CT scanning on admission, and (4) no malignant tumors or autoimmune diseases simultaneously existed. Patients with incomplete clinical information, tuberculosis, asthma, and a previous history of lung surgery were excluded. The whole study cohort was assigned into two groups at random with a ratio of 6:4 (i.e., derivation and internal test cohorts for model development and validation, respectively). Data from an external cohort were obtained from the Suzhou Municipal Hospital. These eligible individuals met the same inclusion criteria as the derivation cohort.

We recorded demographics and laboratory tests from the electronic medical record, and chest CT scans were obtained from a picture archiving and communication system. Patients were regularly followed up every 3 months for 2 years.

The ethical guidelines of the 1975 Declaration of Helsinki were strictly followed. The review board of both institutions approved our study, and informed consent was waived.

### Potential predictors

The BODE index is a simple multidimensional grading system for assessing the life quality of patients with COPD. This score consists of body mass index, airflow obstruction, dyspnea defined by the mMRC questionnaire, and exercise capacity index (25, 26). Previous exacerbation history was defined that patients had at least one COPD exacerbation since the initial diagnosis of COPD (27).

### Outcomes

The clinical outcome of this study is a moderate (requiring antibiotics and/or glucocorticoids) or severe (hospitalization, ICU admission, or death) exacerbation (10).

### CT images acquisition

CT scans were performed using a 64-slice spiral CT (SIEMENS CT scanner, Erlangen, Germany; Philips CT scanner, Cleveland, USA). On admission, all subjects underwent chest CT scans at 120 kVp tube voltage and 200 mA tube current. Volumetric inspiratory and expiratory CT was performed according to a standardized study protocol (28). CT images were reconstructed with a 1-mm section thickness and were standardized before review and evaluation. More information was provided in Supplementary material S1.

### Chest CT image analysis

The function status of the whole lung was quantitatively evaluated by 3D slicer software (version 5.2.2) (29). Quantitative volumetric analysis was conducted based on built-in modules, and individual volumes were calculated, including emphysema [low-attenuation areas (voxels with ≤ −950HU)] (30). Functional lung volume (FLV) is defined as the total volume of ventilated lung tissue. Affected lung volume (ALV) is calculated as the sum of the volume of infiltrated and collapsed lung tissue.

Next, the body composition of all subjects was evaluated using Slice-O-Matic (TomoVision, Magog, Canada, version 5.0) software (31). An axial CT slice at the level of the T4 was selected to measure the total cross-sectional area (CSA) of the pectoralis major and minor muscles. According to the predefined HU threshold, at the level of L1, visceral adipose tissue area (VATA, −150 to −50 HU), subcutaneous adipose tissue area (SATA, −190 to −30 HU), and skeletal muscle area (SM, −29 to 150 HU) were calculated (32, 33). Two clinicians (5- and 8-years of experience in radiology, respectively) independently reviewed and measured all CT indicators when blind to clinical information.

### Model construction and evaluation

This model for predicting COPD exacerbations was developed using the entire derivation dataset. Artificial neural network (ANN), as one of the deep learning algorithms, was introduced for predicting exacerbations in this study. For optimizing the architecture and parameters of models, we got the best parameter combinations of each model using the cross-validated grid-search (5-fold) method with software (Python, version 3.6.2; scikit-learn package, version 0.24). The final models were determined for further evaluation based on the best parameters and selected variables. Internal and external test datasets were used to evaluate the predictive performance of this system. According to the first quartile (25%) and the third quartile (75%) of probabilities from this system, the cohort was divided into low-, medium-, and high-risk groups. More information was provided in Supplementary material S2.

### Statistical analysis

Continuous data were shown as the median ± interquartile range (IQR) and compared using the Mann–Whitney U-test or Student’s t-test as appropriate. Categorical data were compared using the chi-squared test.

Parameters with a possibility threshold of <0.05 based on univariate logistic regression analysis were included in the multivariate logistic regression model for identifying independent predictors associated with COPD exacerbations. We adopted the Spearman correlation coefficient (Rho) to evaluate correlations between independent predictors.

The ROC analysis was performed to evaluate the predictive performance. We compared the differences between ROC curves using the DeLong test. The Kaplan–Meier method and the log-rank test were used to estimate survival without exacerbations.

IBM SPSS version 26.0 was used for data analysis. *p* < 0.05 were considered statistically significant.

## Results

### Patient characteristics

**Figure 1:**
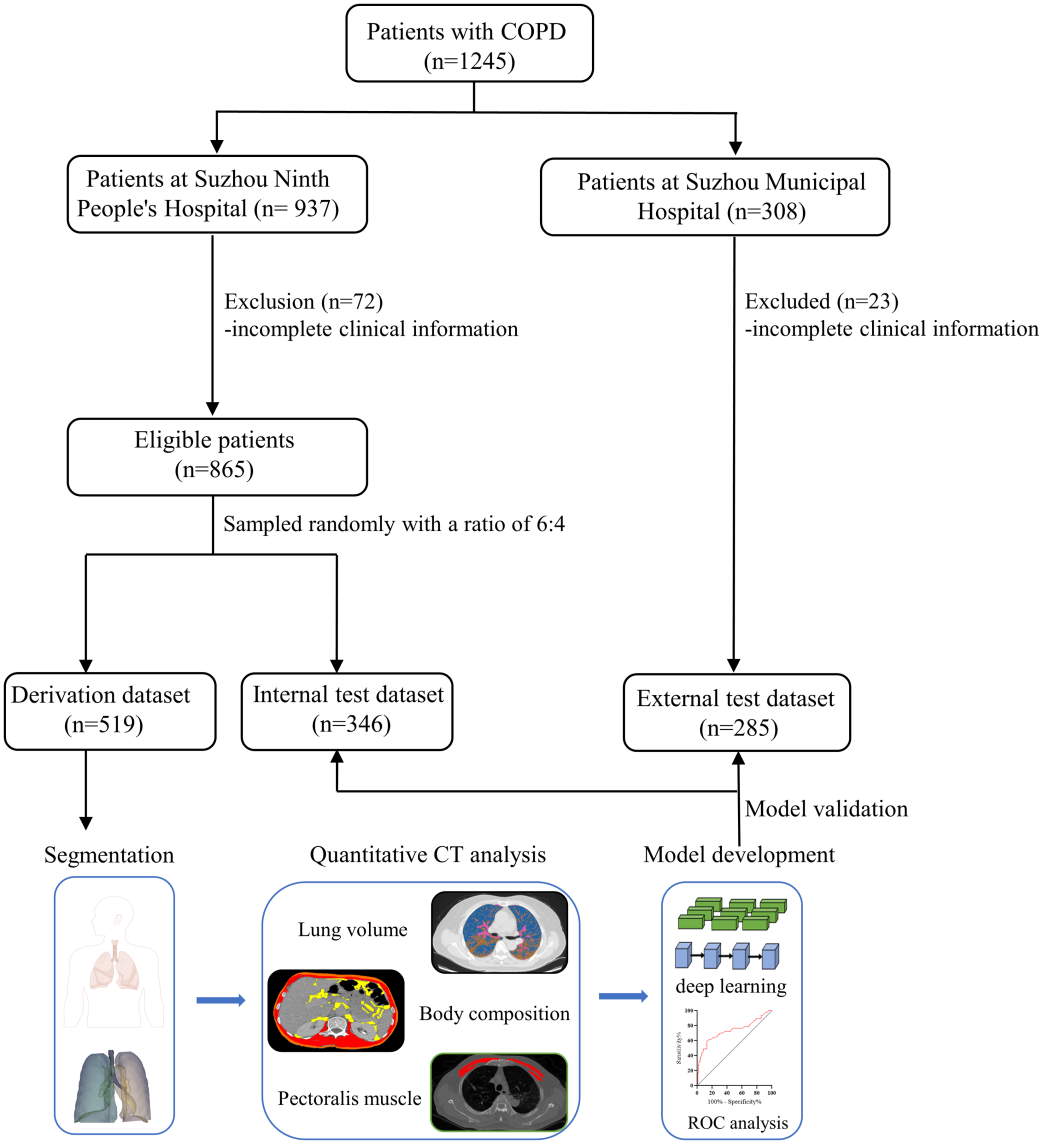
Flow diagram of the study population and model development.

A total of 1,150 eligible patients were enrolled, and the flowchart of the study design is shown in Figure 1. The derivation cohort for model development consisted of 519 patients, and the remaining patients were assigned to internal (*n* = 346) and external test cohorts (*n* = 285).

In the derivation cohort, the median age and body mass index (BMI) were 62 (IQR, 10.5) years and 23.6 (IQR, 4.8), respectively. Patients with ≥2 comorbidities accounted for 71.8%. During a 2-year follow-up, 90 patients (17.3%) suffered from ≥1 exacerbation. Furthermore, we retrospectively enrolled 50 sex- and age-matched healthy controls in this study. The baseline clinical characteristics are summarized in Table 1 and Supplementary Table S1.

**Table 1:** Baseline characteristics.

Variable	Derivation cohort (*n* = 519)	Internal cohort (*n* = 346)	External cohort (*n* = 285)
Age, y^a^	62 (10.5)	59(12)	65(16)
Male, sex^b^	347(66.9)	215(62.1)	192(67.4)
BMI, kg/m^2^^a^	23.6 (4.8)	22.9 (4.2)	22.5(3.9)
No. of comorbidities^b^			
0–1	146(28.2)	113(32.7)	99(34.7)
≥2	373(71.8)	233(67.3)	184(65.3)
SpO2 at admission%^a^	95.3(2.3)	96.3(2.7)	97.8(1.9)
Current smokers^b^	162(31.2)	93(26.9)	32.6(93)
Brinkman index>400^b^	127(24.5)	67(19.3)	71(24.9)
Severe exacerbation history^b^	101(19.5)	59(17.1)	53(18.6)
BODE index^a^	2(2)	1.5(2)	2(2)
GOLD grade^b^			
0	178(40.7)	138(40)	112(39.3)
1	37(11)	51(14.7)	35(12.3)
2	62(33.1)	108(31.2)	105(36.8)
3	106(11.2)	34(9.8)	21(7.4)
4	18(4)	15(4.3)	12(4.2)
Post-bronchodilator FEV_1_% pred^a^	70.5 (17.7)	73.6 (20.1)	65(23.2)
FEV_1_/FVC ratio^a^	55.3 (16·8)	59.8 (21)	49.8(18.9)
CT-derived lung-related parameters^a^			
LAA_950_, %	11.2(10.2)	13.7(11.6)	16.3(9.6)
Functional lung volume/TLC	62.1(21.3)	65.3(18.5)	70.3(15.7)
Affected lung volume /TLC	43(16.9)	37.8(19.3)	46.2(15.1)
Body composition analysis			
VAT, cm^2^	117.9(89.1)	113.2(92.7)	122.3(81.6)
SAT, cm^2^	101.2(97.8)	106.9(105.5)	94(94.4)
SM, cm^2^	97.4(36.3)	92.9(42.7)	89.1(38.8)
Pectoralis muscle, cm^2^	37.2(13.3)	36.4(12.2)	42.5(10.3)
Laboratory tests^a^			
White blood cell count, 10^9^/L	7.6(2.1)	7.9(2.3)	7.3(1.9)
Neutrophil count, 10^9^/L	4.9(1.8)	5.1(2.3)	4.8(2.5)
Lymphocyte count, 10^9^/L	2.1(0.9)	1.9(0.7)	2.2(1.1)
Hemoglobin, g/L	135(23)	131(26)	124(31)
INR	0.98(0.1)	0.99(0.09)	1(0.11)
PLT, 10^9^/L	226(82)	236(91)	219(93)
ALB, g/L	37.1(6.6)	35.2(9.3)	36(9.8)
ALT, U/L	17(7)	18(8)	15(10)
AST, U/L	12(7)	16(12)	17(11)
TBIL, μmol/L	11.8(5.1)	10.4(5.4)	12.5 (6.2)
Cr, μmol/L	64(25)	62(27)	71(32)
PLR score	138(75)	144(67)	129(84)
NLR score	2.7(1.9)	2.5(1.8)	2.6(2.1)
SII score	514.2(450.2)	498.4(397.1)	586(597.3)

a Quantitative values are median (IQR).

b Categorical variables are *n* (%). Comorbidities include hypertension, diabetes, cardiovascular diseases (including coronary heart disease and rheumatic heart disease), cerebrovascular diseases (including cerebral infarction and cerebral thrombosis), pulmonary diseases (including chronic bronchitis and interstitial pneumonia), and chronic renal failure.

BMI, body mass index; BODE, body mass index, airflow obstruction, dyspnea, and exercise capacity index; GOLD, Global Initiative for Chronic Obstructive Lung Disease; FEV1, forced expiratory volume in 1 s. FVC, forced vital capacity; LAA_950_, low-attenuation area (voxels with ≤ −950HU); TLC, total lung capacity; VAT, visceral adipose tissue area; CSA, cross-sectional area; SAT, subcutaneous adipose tissue area; SM, skeletal muscle area; INR, international normalized ratio; PLT, platelet count; ALB, albumin; ALT, alanine aminotransferase; AST, aspartate aminotransferase; TBIL, total bilirubin; Cr, creatinine. PLR, platelet to lymphocyte ratio; NLR, Neutrophil to lymphocyte ratio; SII, systemic immune-inflammation index.

### Identification of independent risk factors

In the derivation cohort, the univariate logistic regression analysis revealed that ≥2 comorbidities, history of exacerbation, BODE index, post-bronchodilator FEV1% pred, and several CT-derived parameters were associated with exacerbation events (Table 2). In the multivariate analysis, history of exacerbation (OR, 1.32), BODE index (OR, 1.17), ALV/TLC ratio (OR, 1.61), VAT (OR, 1.18), and pectoralis muscle CSA (OR, 0.95) were associated significantly with exacerbation events (Table 2). A correlation map among different predictors is shown in Supplementary Figure S1 (see Table 3).

**Table 2:** Univariate and multivariate logistic analyses to identify predictors of COPD exacerbations in the derivation cohort.

Variable	Univariate analysis		Multivariate analysis	
	Odds ratio	*p*-value	Odds ratio	*p*-value
≥2 comorbidities	1.23	0.043		
History of exacerbation	1.29	0.030	1.26	0.027
BODE index	1.23	0.021	1.29	0.013
Post-bronchodilator FEV_1_% pred	0.93	<0.001		
CT-derived parameters				
Functional lung volume/TLC	0.92	0.014		
Affected lung volume /TLC	1.52	0.009	1.66	<0.001
VAT	1.14	0.018	1.37	0.002
SM	0.891	0.038		
Pectoralis muscle CSA	0.96	0.007	0.85	<0.001
Laboratory tests				
White blood cell count	1.06	0.041		
Neutrophil count	1.22	0.015		
Lymphocyte count	1.18	0.024		
PLR score	1.09	0.032		
NLR score	1.21	0.018		

COPD, chronic obstructive pulmonary disease; BMI, body mass index; BODE, body mass index, airflow obstruction, dyspnea, and exercise capacity index; FEV1, forced expiratory volume in 1 s. FVC, forced vital capacity; LAA_950_, low-attenuation area (voxels with ≤ −950HU); TLC, total lung capacity; VAT, visceral adipose tissue area; SAT, subcutaneous adipose tissue area; SM, skeletal muscle area; CSA, cross-sectional area.

**Table 3:** Predictive performance of the DLS for prediction of COPD exacerbations in all cohorts.

Model	Derivation cohort		Internal cohort		External cohort	
	AUC (95% CI)	*p* value	AUC (95% CI)	*p* value	AUC (95% CI)	*p* value
DLS	0.90 (0.86, 0.95)	<0.001	0.88 (0.82, 0.92)	<0.001	0.86 (0.81, 0.89)	<0.001
Exacerbation history	0.79 (0.71, 0.82)	0.012	0.76 (0.71, 0.82)	0.023	0.75 (0.73, 0.80)	0.029
BODE index	0.81 (0.76, 0.84)	0.008	0.80 (0.73, 0.86)	0.008	0.78 (0.75, 0.83)	0.016

COPD, chronic obstructive pulmonary disease; DLS, deep learning system; BODE, BMI, airflow obstruction, dyspnea, and exercise capacity index; AUC, area under of ROC curve; CI, confidence interval.

### Predictive performance of the DLS

**Figure 2:**
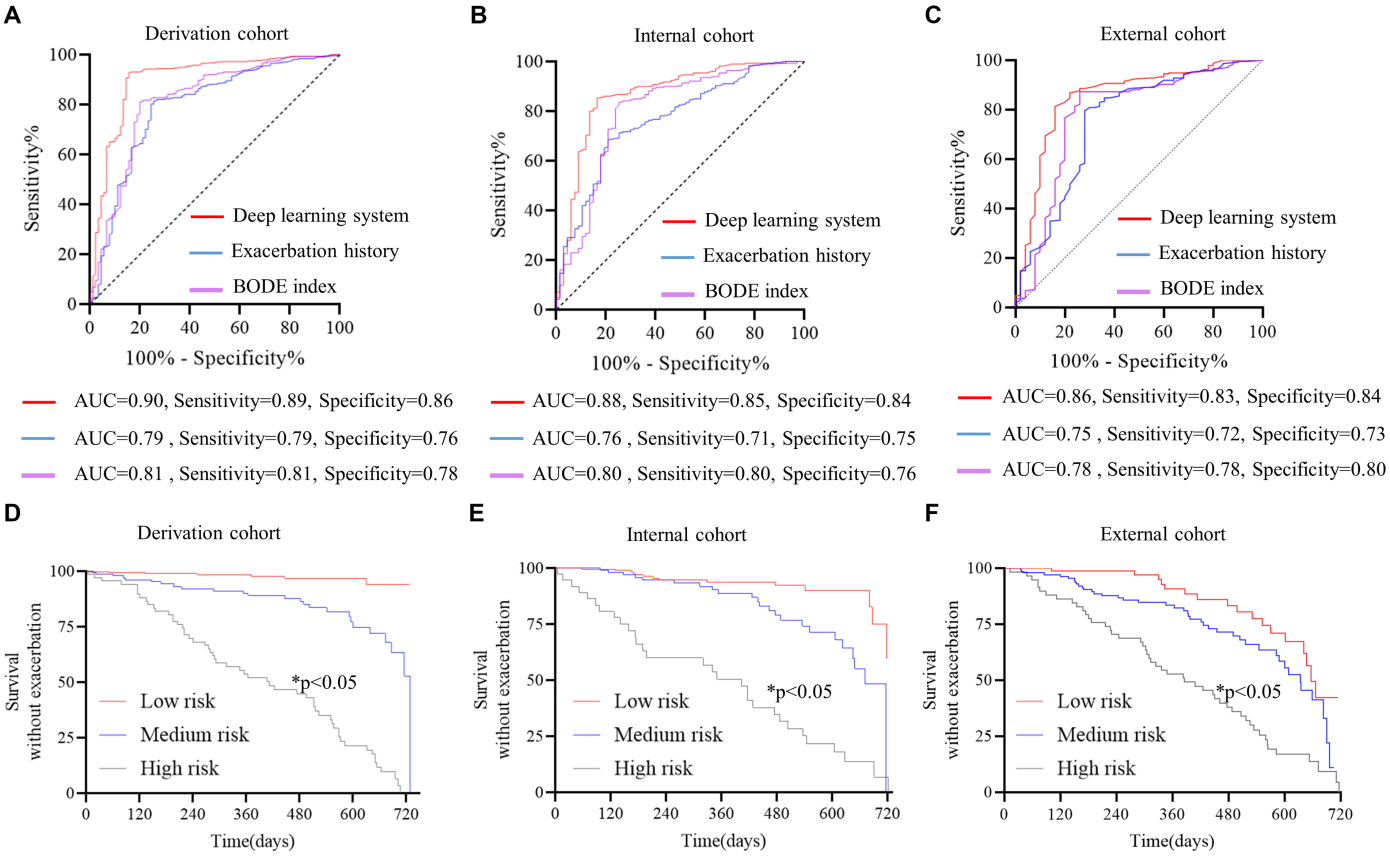
Discriminative performance of the deep learning system (DLS) for predicting future severe acute COPD exacerbations. **(A–C)** The red curve represents DLS, the blue curve represents exacerbation history, and the purple curve represents the BODE index. The DeLong test was used to compare ROC curves, and AUCs were higher for the DLS than exacerbation history and the BODE index (DeLong test *p* < 0.05 for all). Survival analysis without exacerbation **(D–F)** all patients were stratified according to prediction possibility from the DLS. The Kaplan–Meier method and log-rank test were used to estimate the survival without exacerbations. Patients at high risk were prone to exacerbation (*p* < 0.05 for all). The red curve represents patients at low risk, the blue curve represents patients at medium risk, and the gray curve represents patients at high risk.

The DLS was developed for predicting at least one exacerbation episode in 2 years, based on clinical data (history of exacerbation and BODE index) and CT-derived radiomic parameters (ALV/TLC ratio, VAT, and pectoralis muscle CSA). Compared with the history of exacerbations and BODE index, this system showed the best predictive performance with an AUC of 0.90 (95% CI, 0.86–0.94) in the derivation cohort, 0.88 (95%CI, 0.82–0.92) in the internal cohort, and 0.86 (95%CI, 0.81–0.89) in the external cohort (Figures 2A–C, Table 3). The AUC values of the DLS in derivation and test cohorts were significantly higher than the history of exacerbations and BODE index using the DeLong test (*p* < 0.05).

Patients were stratified by probabilities from the DLS in the derivation cohort. In the survival analysis, patients with a higher risk were prone to pulmonary exacerbations (*p* < 0.05) (Figures 2D–F).

### Risk stratification of subgroups

**Figure 3:**
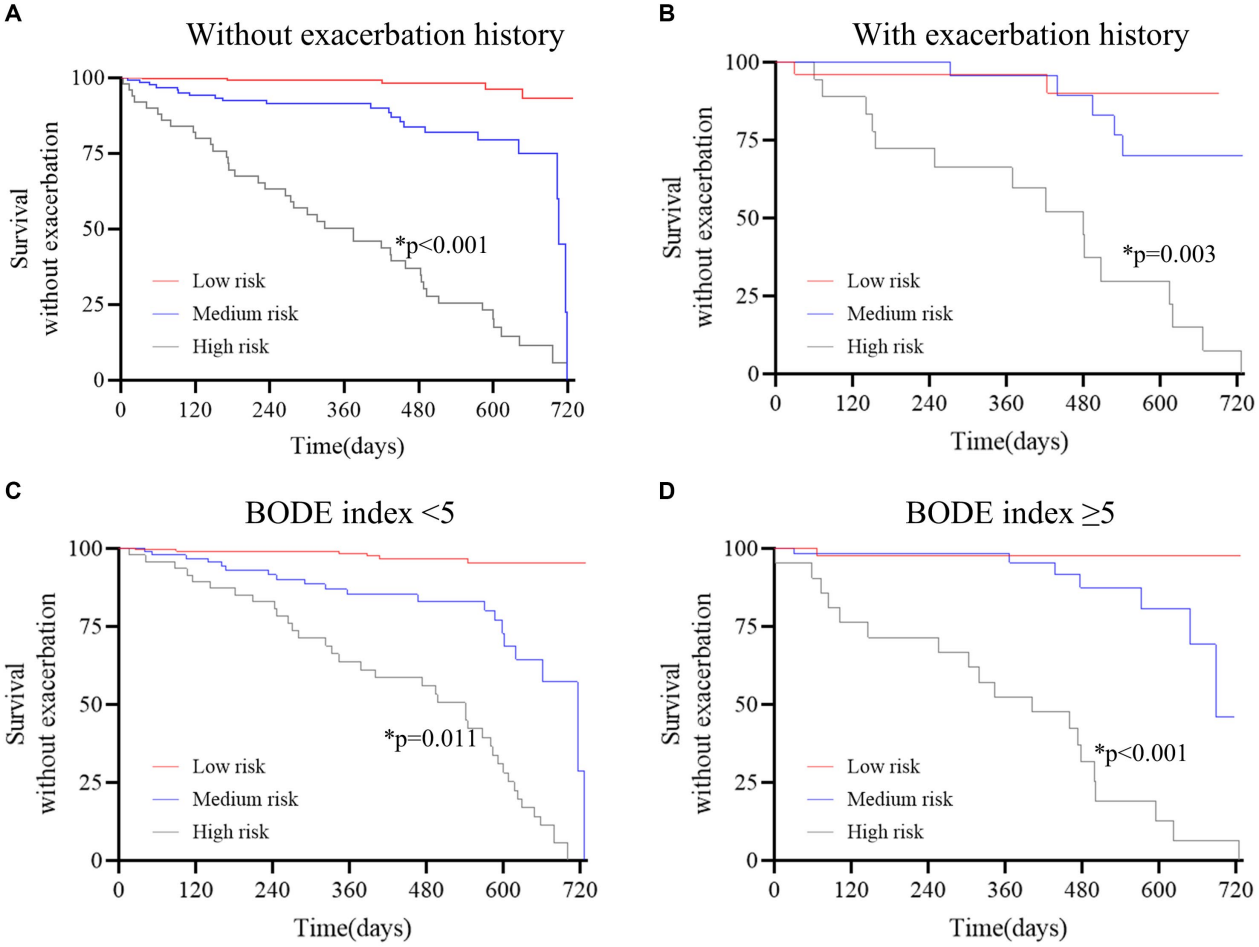
Survival curves stratified by the deep learning system in the derivation cohort. **(A)** without exacerbation history; **(B)** with exacerbation history; **(C)** BODE index<5; D, BODE index≥5. The red curve represents patients at low risk, the blue curve represents patients at medium risk, and the gray curve represents patients at high risk. The Kaplan–Meier method and the log-rank test were used to estimate survival without exacerbations. The results revealed that patients at high risk were prone to exacerbation in all subgroups (*p* < 0.05 for all).

To evaluate the ability of the DLS to reclassify the risk of COPD patients, the survival analysis was performed in certain patient subgroups (Figure 3). Additional discrimination of the DLS for COPD exacerbations was determined, such as in patients with a BODE index of ≥5.

## Discussion

In the present study, the multivariate analysis revealed that CT-derived high ALV/TLC, high VAT CSA, and low pectoralis muscle CSA were independent risk factors causing COPD exacerbations. Remarkably, the DLS was developed and validated to identify individuals at risk of COPD exacerbations during 2-year follow-ups based on clinical data and CT-derived biomarkers. This system significantly outperformed conventional scores (exacerbation history and BODE index), with an AUC value of 0.88. Furthermore, this tool showed potential for stratifying patients by the probability of exacerbations and offered evidence to make individual care plans.

Recently, multiple studies reported that CT scan-derived body composition parameters (muscle and adipose tissue mass and distribution) were correlated with adverse clinical outcomes such as lung cancer death, adverse cardiovascular events, and fragility fractures (34, 35). Our study showed a close correlation between high VAT and future COPD exacerbations. Obesity, and abdominal VAT, in particular, is an important and distinguishing characteristic in COPD patients, and that may be due to adipose tissue function alteration (36–38). In addition, pectoralis muscle CSA could be used to evaluate the clinically relevant muscle mass (39). Generally, low muscle mass represents a decrease in the reserve of multiple physiological systems (40). This could lead to worst outcomes, including COPD exacerbations. Moreover, high ALV/TLC was found to be significantly linked to COPD exacerbations. This may be attributed to inflammatory cell infiltration and the collapse of lung tissue structure (41).

To the best of our knowledge, a history of exacerbation and the BODE index were widely used indicators for predicting COPD (10, 42, 43). However, exacerbation history has some limitations, such as inherent recall bias and unexplained underlying mechanisms. These affect the application of this predictor in patients without exacerbation history. Similarly, the BODE index consists of four variables, including the variable exercise capacity, and its predictive performance is unsatisfactory. The DLS in our study might serve a pivotal role in overcoming these limitations.

Furthermore, our data demonstrated that patients with higher risk stratified by the DLS were susceptible to the exacerbations of COPD. Clinicians can identify high-risk patients using the DLS, thereby making adequate antimicrobial therapy. Low-risk individuals only receive a standard of care. This might guard against the over-medicalization. In addition, certain patient subgroups may benefit from this risk assessment tool, such as those with no exacerbation history/BODE<5, yet high risk identified by the DLS.

However, our study has some limitations. First, this study is a retrospective study, and the generalizability should be validated in a large external dataset. Second, the analysis of CT scan-derived parameters requires experienced clinicians, and it might affect the practical use of the DLS in the clinic. A fully automated tool should be devised for image analysis. Third, the underlying relationship between these CT biomarkers and future exacerbations requires further investigation. Finally, although respiratory questionnaires for clinical symptom evaluation have some limitations, the potential value of these scores in predicting COPD exacerbations should be investigated in future studies.

## Conclusion

High VAT, low pectoralis muscle CSA, and high ALV/TLC were independent risk factors of COPD exacerbations. We developed and externally validated the DLS to identify individuals at risk for future exacerbations. This system is expected to improve healthcare quality and reduce medical costs.

## Data availability statement

The raw data supporting the conclusions of this article will be made available by the authors, without undue reservation.

## Ethics statement

The studies involving humans were approved by the ethics committee of Suzhou Ninth Hospital Affiliated to Soochow University. The studies were conducted in accordance with the local legislation and institutional requirements. The ethics committee/institutional review board waived the requirement of written informed consent for participation from the participants or the participants’ legal guardians/next of kin because the nature of the retrospective study.

## Author contributions

JW: Validation, Supervision, Resources, Writing – review & editing, Writing – original draft, Visualization, Methodology, Investigation, Conceptualization. YL: Software, Project administration, Writing – review & editing, Writing – original draft, Visualization, Investigation, Conceptualization. SD: Methodology, Funding acquisition, Formal analysis, Data curation, Writing – review & editing, Software, Project administration, Investigation. LW: Supervision, Resources, Conceptualization, Writing – review & editing, Software, Project administration, Investigation, Data curation. XS: Writing – original draft, Visualization, Methodology, Funding acquisition, Writing – review & editing, Investigation, Conceptualization.
