# Supplementary material for: Predicting COPD exacerbations based on quantitative CT analysis: an external validation study
Source: Front Med (Lausanne). 2024 Jun 12;11:1370917. doi: 10.3389/fmed.2024.1370917 (PMC11199769; doi:10.3389/fmed.2024.1370917)
Supplement: Supplementary file 1 [file Data_Sheet_1.DOCX]

# Supplementary Data

1.1 **Supplementary S.1. Image acquisition**

All Chest CT images were obtained using a 64-slice spiral CT (PHILIPS, SIEMENS) during breath-hold. The subjects underwent two volumetric chest CT examinations: one at full inspiration (TLC) and one at the end of a normal expiration (FRC). These scans were reconstructed with a slice thickness of 1 mm. The near-isotropic voxels were achieved. Two manufacturers were used in the study. Inspiratory scans were acquired at 200 mAs and expiratory scans, at 50 mAs; all scans were acquired at 120 kVp. CT dose modulation and IV contrast agents were not used for this study. The average effective tube current–exposure time product was 46 mAseff for expiratory scans and 180 mAseff for inspiratory scans, and pitch values ranged from 0.9 to 1.4 depending on the manufacturer and scanner model. The CT data was saved in the format of DICOM.

1.2 **Supplementary S.2. Model parameters**

The optimal parameter combinations of our model are described as follows (determined by grid search):

Deep learning (artificial neural network), epochs, 21, hidden layers, 11 optimizer, Adam; batch size of 10; learning rate, 0.01; Dropout, 2.

**Supplementary Table 1. Baseline characteristics of derivation cohort**

| Variables | Control (n=50) | Stable  (n=429) | exacerbation (n=90) | P value |
| --- | --- | --- | --- | --- |
| Age, y ^a^ | 61(8) | 61(8.5) | 63(12) | 0.368 |
| Male gender ^b^ | 32(64) | 279(65) | 68(76) | 0.073 |
| BMI, kg/m^2^ ^a^ | 23.1(3.9) | 23.4(4.2) | 23.8(3.9) | 0.590 |
| ≥2 comorbidities ^b^ | 22(44) | 292(68.1) | 81(90) | **0.043** |
| SpO2 at admission% ^a^ | 98(2.2) | 95.5(2.7) | 94.7(3.1) | 0.851 |
| Current smokers ^b^ | 17(32) | 133(31) | 29(32.2) | 0.745 |
| Brinkman index>400 ^b^ | 6(12) | 120(21.9) | 131(36.7) | 0.067 |
| Severe exacerbation history ^b^ | NA | 37(8.6) | 64(71.1) | **0.030** |
| BODE index ^a^ | NA | 2(2) | 4(2) | **0.021** |
| Postbronchodilator FEV_1_ % pred ^a^ | NA | 75.2 (15.4) | 63.1 (19.6) | **<0.001** |
| FEV_1_/FVC ratio ^a^ | NA | 57.8(12.5) | 52.2 (18.3) | 0.452 |
| CT-derived lung-related parameters ^a^ |  |  |  |  |
| LAA950, % | 6.5(8.7) | 10.9(12.4) | 12.1(8.5) | 0.333 |
| Functional lung volume/TLC | 83(12.2) | 64.2(17.7) | 55.1(14.3) | **0.014** |
| Affected lung volume /TLC | 22(7.4) | 39.8(16.5) | 48.4(21.2) | **0.009** |
| Body composition analysis |  |  |  |  |
| VAT, cm^2^ | 102.7(72.2) | 112.7(79.5) | 133.6(85.1) | **0.018** |
| SAT, cm^2^ | 95.1(82.5) | 98.3(62.6) | 109.1(97.2) | 0.517 |
| SM, cm^2^ | 109(31.9) | 102.4(34.7) | 89.3(23.5) | **0.038** |
| Pectoralis muscle, cm^2^ | 45.2(11.4) | 39.6(14.2) | 32.3(14.4) | **0.007** |
| Laboratory tests ^a^ |  |  |  |  |
| White blood cell count, 10^9^/L | 6.3(1.7) | 7.2(1.9) | 8.2(2.3) | **0.041** |
| Neutrophil count, 10^9^/L | 3.5(1.4) | 4.7(2.1) | 6.2(1.9) | **0.015** |
| Lymphocyte count, 10^9^/L | 2.5(1) | 2.1(1.1) | 1.6(0.9) | **0.024** |
| Hemoglobin, g/L | 141 (25) | 137(23) | 132(28) | 0.924 |
| INR | 0.96(0.06) | 0.99(0.08) | 0.99(0.1) | 0.624 |
| PLT, 10^9^/L | 242(67) | 219(74) | 228(82) | 0.841 |
| ALB, g/L | 40.1(6.6) | 37.7(8.4) | 35(7.6) | 0.424 |
| ALT, U/L | 14(6) | 18(6) | 16(8) | 0.766 |
| AST, U/L | 10(5) | 11(10) | 14(9) | 0.691 |
| TBIL, μmol/L | 9.7(2.5) | 10.9(6.1) | 13.2 (5.4) | 0.597 |
| Cr, μmol/L | 62(30) | 59(12) | 68(25) | 0.351 |
| PLR score | 98(69) | 102(59) | 141(71) | **0.032** |
| NLR score | 1.8(1.1) | 2.5(2.1) | 3.7(2.9) | **0.018** |
| SII score | 472.1(375.5) | 504(426.2) | 556(513.9) | 0.216 |

Note: a. Quantitative values are Median (IQR); b. Categorical variables are n (%). Comorbidities include hypertension, diabetes, cardiovascular diseases (including coronary heart disease, and rheumatic heart disease), cerebrovascular diseases (including cerebral infarction and cerebral thrombosis), pulmonary diseases (including chronic bronchitis and interstitial pneumonia), chronic renal failure.

Abbreviations: BMI, body mass index; BODE, body mass index, airflow obstruction, dyspnea, and exercise capacity index; FEV1, forced expiratory volume in 1 s. FVC, forced vital capacity; LAA_950_, low-attenuation area (voxels with ≤ −950HU); TLC, total lung capacity; VAT, visceral adipose tissue area; SAT, subcutaneous adipose tissue area; SM, skeletal muscle area; CSA, cross-sectional area; INR, international normalized ratio; PLT, platelet count; ALB, albumin; ALT, alanine aminotransferase; AST, aspartate aminotransferase; TBIL, total bilirubin; Cr, creatinine. PLR, platelet to lymphocyte ratio; NLR, Neutrophil to lymphocyte ratio; SII, systemic immune-inflammation index.


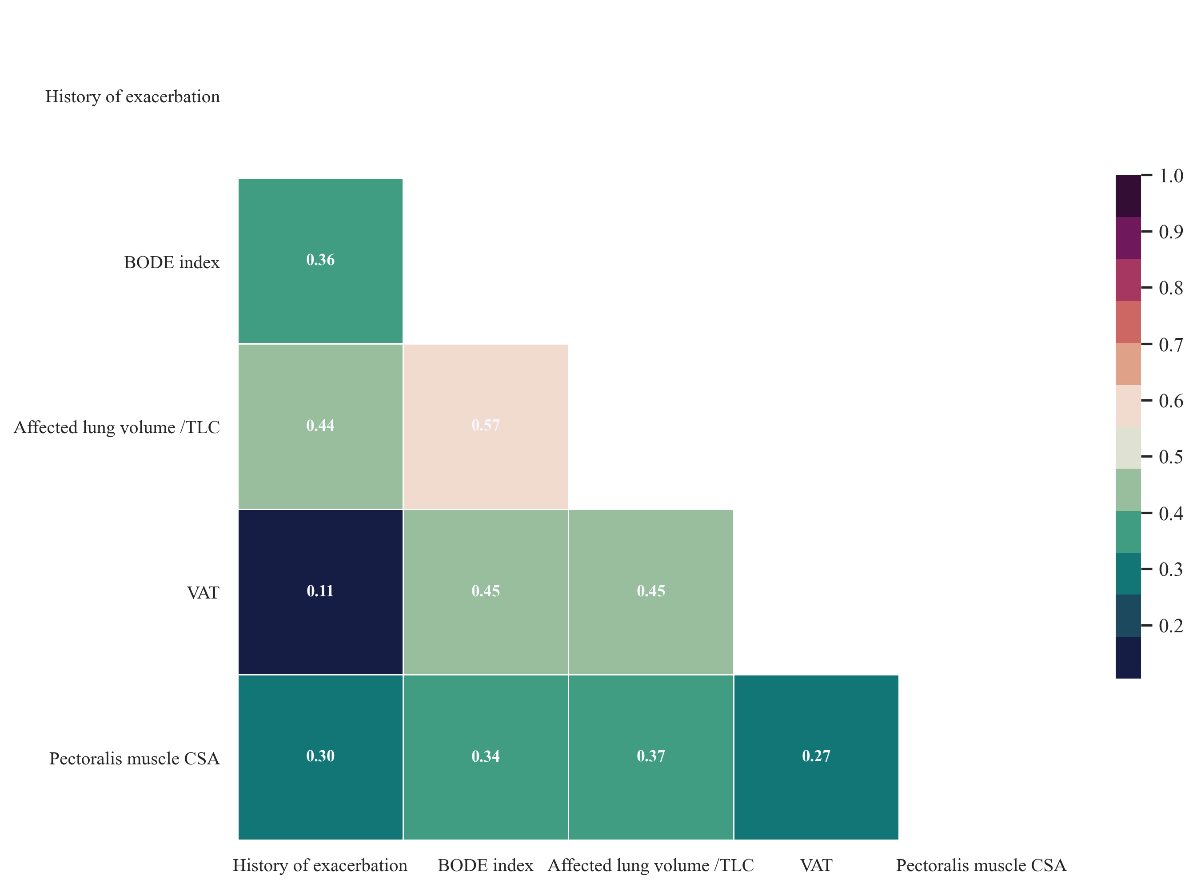


Supplement Figure 1. Correlation map among independent predictors. We adopted spearman correlation coefficient (**|**Rho**|**) to evaluate correlations among different predictors. The colors represent the magnitude of correlation coefficient.

Abbreviations: BODE, body mass index, airflow obstruction, dyspnea, and exercise capacity index; TLC, total lung capacity; VAT, visceral adipose tissue area; CSA, cross-sectional area.
